# Acute Systemic White Blood Cell Changes following Degenerative Cervical Myelopathy (DCM) in a Mouse Model

**DOI:** 10.3390/ijms231911496

**Published:** 2022-09-29

**Authors:** Antigona Ulndreaj, Ariel Ávila, James Hong, Cindy Zhou, Michael G. Fehlings, Pia M. Vidal

**Affiliations:** 1 Lunenfeld-Tanenbaum Research Institute, Mount Sinai Hospital, Toronto, ON M5G 1X5, Canada; 2 Developmental Neurobiology Unit, Biomedical Science Research Laboratory, Basic Sciences Department, Faculty of Medicine, Universidad Católica de la Santísima Concepción, Concepción 4090541, Chile; 3 Department of Genetics and Development, Krembil Research Institute, University Health Network, Toronto, ON M5G 2C4, Canada; 4 Institute of Medical Science, University of Toronto, Toronto, ON M5S 1A1, Canada; 5 Spinal Program, University Health Network, Toronto Western Hospital, Toronto, ON M5T 2S8, Canada; 6 Neuroimmunology and Regeneration of the Central Nervous System Unit, Biomedical Science Research Laboratory, Basic Sciences Department, Faculty of Medicine, Universidad Católica de la Santísima Concepción, Concepción 4090541, Chile

**Keywords:** degenerative cervical myelopathy (DCM), white blood cells, innate and adaptive immune system

## Abstract

Degenerative cervical myelopathy (DCM) is caused by age-related degeneration of the cervical spine, causing chronic spinal cord compression and inflammation. The aim of this study was to assess whether the natural progression of DCM is accompanied by hematological changes in the white blood cell composition. If so, these changes can be used for diagnosis complementing established imaging approaches and for the development of treatment strategies, since peripheral immunity affects the progression of DCM. Gradual compression of the spinal cord was induced in C57B/L mice at the C5-6 level. The composition of circulating white blood cells was analyzed longitudinally at four time points after induction of DCM using flow cytometry. At 12 weeks, serum cytokine levels were measured using a Luminex x-MAP assay. Neurological impairment in the mouse model was also assessed using the ladder walk test and CatWalk. Stepping function (* *p* < 0.05) and overground locomotion (*** *p* < 0.001) were impaired in the DCM group. Importantly, circulating monocytes and T cells were affected primarily at 3 weeks following DCM. T cells were two-fold lower in the DCM group (*** *p* < 0.0006), whereas monocytes were four-fold increased (*** *p* < 0.0006) in the DCM compared with the sham group. Our data suggest that changes in white blood cell populations are modest, which is unique to other spinal cord pathologies, and precede the development of neurobehavioral symptoms.

## 1. Introduction

Degenerative cervical myelopathy (DCM) is an overarching term used to describe the most common non-traumatic forms of cervical spinal cord myelopathy affecting the elderly population [1,2]. It is caused by age-related degeneration of the cervical spine, leading to chronic compression of the spinal cord accompanied by symptoms in the upper and lower extremities, including weakness, numbness, gait deficits and pain [3]. The current treatment for DCM consists of surgical decompression (herein referred to as decompression) [2,4]. Due to the chronic age-related progressive nature of compression in DCM, the pathophysiology of the disease is quite different from traumatic injuries to the spinal cord which have an acute onset [2,5]. For example, traumatic spinal cord injury (SCI) is accompanied by acute systemic hematological changes and neuroinflammation, which affect natural recovery after injury [1,6,7,8]. However, while neuroinflammation is known to occur locally at the spinal cord level in non-traumatic injuries such as DCM [9,10,11], it is unknown whether DCM presents with changes in the peripheral immune response. Furthermore, there are gaps in our understanding of the disease progression. Specifically, the extent to which the neuroinflammatory response during DCM is correlated with systemic hematological changes remains unknown, although this has been observed in other chronic central nervous system (CNS) pathologies such as multiple sclerosis (MS) [12]. Filling this knowledge gap holds great clinical promise, from disease diagnosis and prognostication to creating personalized immunomodulatory alternatives for the treatment of DCM (i.e., in patients undergoing non-operative management) based on the patient’s systemic immune profile, as accomplished in the field of traumatic SCI [13,14,15].

In the present study, we examine the temporal profile of circulating white blood cells during the progression of DCM in a clinically relevant mouse DCM model induced at the cervical level. We used flow cytometry to assess changes in the frequency of T cells, granulocytes and monocytes compared with sham and age-matched naive animals. We found that, unlike previous reports in traumatic SCI and MS showing profound changes in the systemic white blood cell composition [7,12,16], DCM causes white blood cell changes at the beginning of DCM development (3 to 6 weeks post-DCM induction), and modest changes in T cells when neurological symptoms are fully present (12 weeks post-DCM induction). Furthermore, no significant changes were found in the systemic production of selected cytokines at 12 weeks post-DCM induction.

## 2. Results

### 2.1. The DCM Model Presents with Detectable Clinically Relevant Neurological Deficits

**Figure 1:**
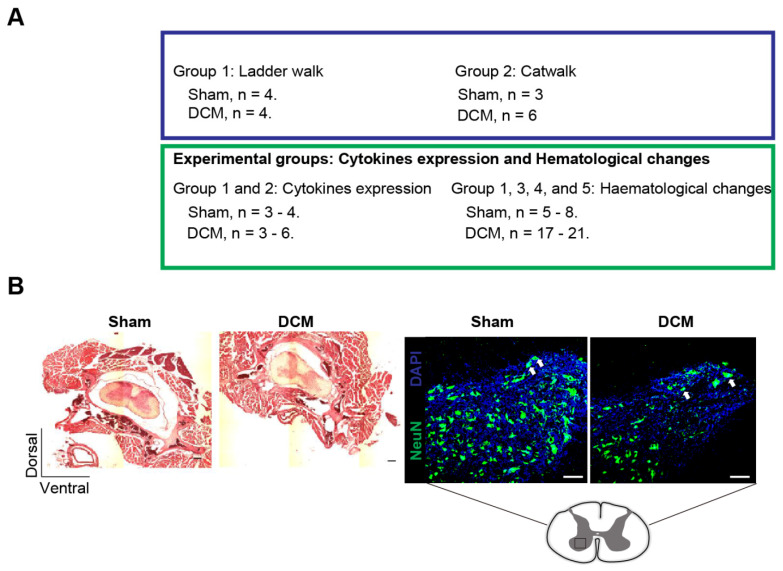
Experimental design and neurological testing. (**A**) Scheme of the experimental design indicating experimental groups and cohorts used for behavioral assessments, hematological changes, and cytokines expression. (**B**) Representative HE-stained coronal sections of the calcified C5-6 area of sham and DCM animals (**left panel**) and NeuN^+^ cells (**right panel**) for the ventral horns. A spinal cord schematic depicting the area of the representative image is shown below. Arrows indicate NeuN^+^ cells. Scale bars = 125 μm for HE images and 500 μm for NeuN staining.

To characterize systemic changes in the blood during the progression of DCM, we designed a randomized, blinded experiment where animals were divided into two groups that received either a sham or DCM surgery (Figure 1A). Using magnetic resonance imaging (MRI) we have previously shown that there is a 47% compression ratio in the mouse DCM model at 12 weeks post DCM-induction [11]. Representative histological hematoxylin and eosin (HE)-stained coronal spinal cord sections of sham and DCM groups at 12 weeks after DCM-induction confirmed spinal cord compression (Figure 1B). Furthermore, these representative images of sham and DCM at 12 weeks after DCM showed a decrease of NeuN^+^ cells in the mouse DCM model (Figure 1B), resembling previously published observations from post-mortem patients with DCM [9]. Previous studies from our group have shown a four-fold loss in the cervical motoneurons in the DCM group versus sham [17,18].

**Figure 2:**
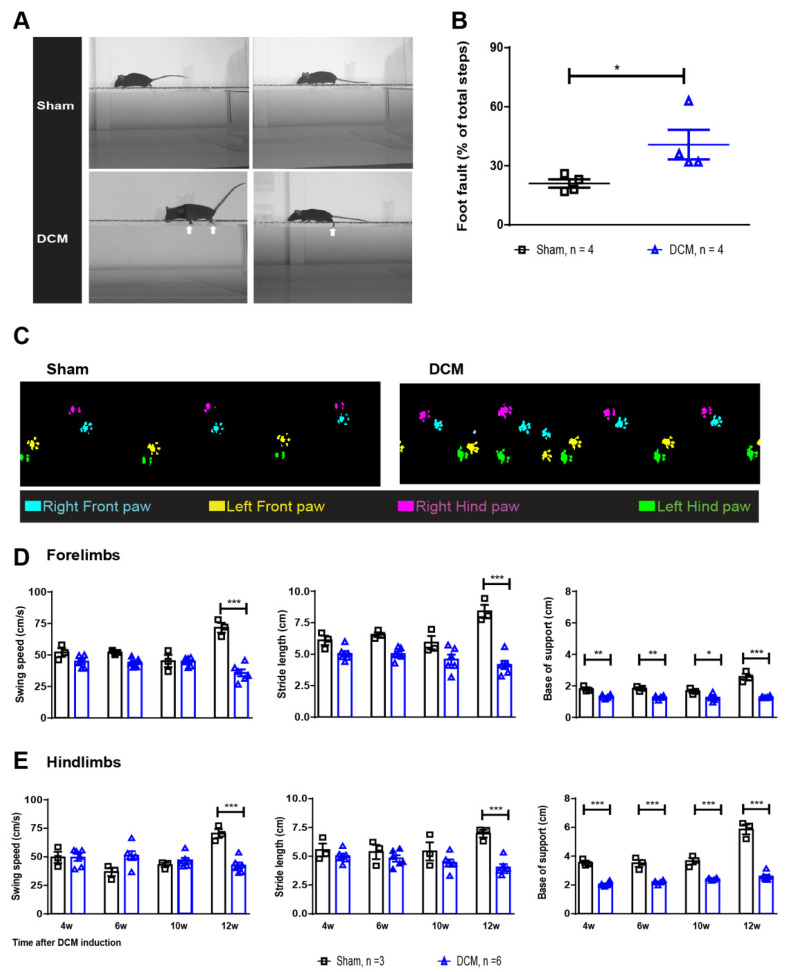
Neurobehavioral impairment following DCM induction in the mouse model. (**A**). Representative images of sham and DCM animals performing the horizontal ladder walk test at 12 weeks post-DCM induction. The arrows represent foot fault in fore- and hindlimbs. (**B**) The average percent (%) foot faults were significantly increased in the DCM group compared with the sham group (* *p* < 0.05, Mann-Whitney U test). Sham = (week 12) = 4; DCM = (week 12) = 4. (**C**) Representative CatWalk footprints from sham and DCM mice. (**D**,**E**) CatWalk results showed a significant decrease in both forelimb and hindlimb overground locomotion. Swing speed and stride length were impaired at 12 weeks after DCM-induction (*** *p* < 0.001, Two-Way ANOVA, Tukey post-hoc), whereas base support deterioration was observed from week 4 onward (* *p* < 0.05; ** *p* < 0.01; *** *p* < 0.001, Two-Way ANOVA, Tukey post-hoc). Data are presented as mean ± SEM.

Additionally, and similar to humans, the DCM mouse model presents with a marked impairment in gait [11,19], as well as in forelimb and hindlimb stepping function. Here the animals with DCM showed a two-fold increase in foot faults during the horizontal ladder walk test compared to the sham group (Figure 2A,B, * *p* < 0.05), indicating detectable neurological deficits related to stepping function. Overground locomotion assessments showed impaired gait in the DCM group. Specifically swing speed and stride length in both forelimbs and hindlimbs were significantly worsened in the DCM group compared with the sham group (Figure 2C–E, *** *p* < 0.001). Furthermore, both forelimb and hindlimb base of support were significantly reduced in the DCM group at all time points assessed (4 w, 6 w, 10 w, and 12 weeks; Figure 2C,D, * *p* < 0.05; ** *p* < 0.01; *** *p* < 0.001).

### 2.2. Monocytes and T Cells Are Affected during the First Weeks Post-DCM Induction

**Figure 3:**
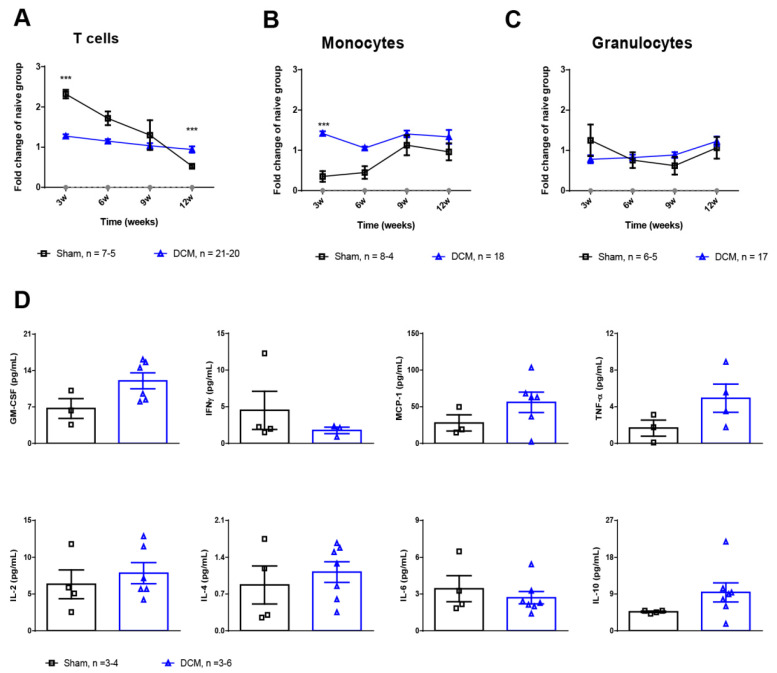
Systemic cellular and cytokine changes after DCM. Flow cytometric quantification of the frequency of circulating T cells (CD3^+^) (**A**) monocytes (Ly6C^+^CD11b^+^Ly6G^−^) (**B**) and granulocytes (Ly6G^+^CD11b^+^Ly6C^−^) (**C**) at 3, 6, 9 and 12 weeks after DCM induction, as compared to the sham group. All values are reported as fold-change relative to age-matched naïve mice. Asterisks indicated differences between groups within each time point. Naive = 6; Sham = 4–8; DCM = 17–21. (**D**) Quantitation of selected inflammatory and anti-inflammatory cytokines in serum at 12 weeks after DCM induction using a Luminex xMAP assay. Data are presented as mean ± SEM. Sham = 3–4; DCM = 3–6.

Overall, fold-changes in CD3^+^ T Lymphocytes (herein referred to as T cells), as compared to matched naïve mice, were significantly different between the DCM and sham group [Mixed effects model, compression × time effect **** *p* < 0.0001, F(3, 70) = 18.93)] (Figure 3A). Particularly, T-cells fold changes between the groups were different at 3 and 12 weeks post-compression (Sidak’s multiple comparison test, *** *p* = 0.0006, *** *p* = 0.0005, respectively). While at 3 weeks, T-cells fold changes were lower in the DCM group compared to shams, by 12 weeks they were higher than in the sham group. These differences between the two groups are most likely driven by the more pronounced decline of T-cells in the sham group compared to the DCM group; by 12 weeks T-cells declined by a factor of 4 in shams compared to a 1.5-fold decrease in the DCM group (Sidak’s multiple comparison test, *** *p* = 0.0002, *** *p* = 0.0005, respectively).

To identify monocytes, we gated the population of Ly6C^+^CD11b^+^Ly6G^−^ cells. Overall, fold-changes in monocytes were significantly different between the groups [Mixed effects model, compression x time effect * *p* = 0.0497, F(3, 56) = 2.775)] (Figure 3B). Particularly at 3 weeks post-compression, monocytes frequencies were higher in the DCM group compared to their sham counterparts (Sidak’s multiple comparison test, *** *p* = 0.0006), while these differences normalized at subsequent time points. Contrary to T-cells, monocytes remained stable across all time points within each group.

Last, fold changes in granulocytes (Ly6G^+^CD11b^+^Ly6C^−^ cells) were similar between the groups and across all time points [Mixed effects model, compression x time effect * *p* = 0.982, F(3, 82) = 2.167)]. However, fold changes increased significantly within the DCM group [repeated measures ANOVA, ** *p* = 0.0097, F(2.161, 34.58) = 5.128], whereas in the sham group they remained stable over time (Figure 3C).

To complement the peripheral inflammatory response following DCM, cytokine levels were analyzed at 12 weeks after DCM induction, whereby the levels of 10 selected cytokines were quantitated in a multiplex fashion. The levels of inflammatory cytokines (GM-CSF, MCP-1, TNF-α) were modestly increased in the DCM group compared with the sham group without reaching significance (Figure 3D).

## 3. Discussion

In this study we quantified systemic hematological changes in the innate and adaptive immune system during the progression of DCM using a clinically relevant DCM model in mice [11]. To our knowledge, this is the first study characterizing systemic changes in the immune system during the progression of DCM. The longitudinal experimental design of this study allows one to determine when systemic changes start to develop in DCM, since in patients with DCM this is not possible to determine given the unknown time of disease onset. In addition, major surgical procedures can lead to alterations in the hemodynamic, endocrine and immune functions of the body, where there is an initial activation of the peripheral immune system along with an enhancement of blood flow to muscle, liver and the ischemic organ [20]. This is followed by a phase of depressed immune function and a recovery phase [21]. Thus, longitudinal characterization of the peripheral immune profile in patients with DCM will advance our understanding of the pathophysiology of DCM and will help answer whether modest changes in white blood cell composition are also observed in patients. Thus, the present study marks the beginning to such endeavors where hematological changes could be used for prognostication and guiding appropriate patient care, similar to reports observed in white blood cells changes in both animal models and DCM patients following surgical decompression [22].

In our DCM mouse model, impairment in manual dexterity and locomotor deficits have been observed around 4 weeks after DCM induction [11,19]. In this study, significant deterioration in base support was observed from 4 weeks onward when compared to their sham counterparts. Furthermore, we detected a decrease in T cells early on during the development of DCM, along with a rise in monocytes at 3 weeks post-DCM induction, which is before symptom onset. However, these systemic changes did not last throughout the disease course, unlike immunological changes in the spinal cord, which are more persistent and have been shown to associate with the development of neurological symptoms in patients with DCM and animal models of the disease [9,10,17]. Although these systemic changes are quite unique compared with other CNS injury models, such as SCI, stroke or MS [12,23,24,25,26], decreased numbers of T cells have been reported as part of the normal response after major surgeries in patients [27] as well as following cervical SCI in patients, a rat model of cervical SCI, and a mouse model of surgical decompression for DCM [8,15,28]. Relatedly, we found that sham and DCM animals presented with significant decline in T cell frequencies over time. However, these changes were more pronounced in the sham group compared to the animals with DCM. Given that we normalized circulating white blood cell responses to matched naïve counterparts, these results suggest that the changes observed over time within the sham group are a natural immunological response to the operation procedure of the sham injury, which includes anesthesia, skin incision, muscle injury and hemorrhage. However, the animals with DCM—which underwent the same surgical procedure as their sham counterparts in addition to permanent material implantation—seem to have lost the ability to mount such an immunological response to surgery. While the implications of this observation in DCM remain to be further explored, a similar phenomenon has been observed in patients with traumatic SCI [29] and a rat model of traumatic SCI [8]; whereby SCI was associated with a less drastic alteration of peripheral T-cell frequencies during the chronic phase of the disease, compared to matched able-bodied persons or sham controls, respectively. Of note, at 12 weeks following DCM T cells are increased. This could be partially explained as a normal aging response or due to the expansion of a specific pathological T cell subpopulation, as reported in other immune-related pathologies [30,31].

Furthermore, experimental animal models of DCM have shown increased levels of cytokines, such as TNF-α, IL-6, IL-13, IL-10, CCL-2, and IL-4 within the spinal cord compared with the control group [10]. These cytokines can contribute to the development of neuroinflammation, both at the spinal cord level or systemically. At the systemic level, increased serum IL-6 levels have been correlated with symptom severity in DCM patients [32]. To our knowledge, there is no information about cytokine production at the systemic level in animal models of the pathology. In the current study, non significant differences were found between the sham and the DCM groups. This discrepancy could be attributed to the different animal models used, spontaneous calcified deposits (twy/twy model) versus implantation of an aromatic polyether material, as well as the lesion level C1-2 versus C5-6.

A previous study has suggested that hematologic changes observed within the first week following traumatic SCI in patients may be a result of post-traumatic decentralization of the autonomic nervous system [28]. During aging certain T cells subsets accumulate in the spleen and peripheral lymph nodes [33]. For example regulatory T cells (T cells, CD25^low^Foxp3^+^) accumulate, correlating with lower IL-2 levels [34]. Similarly the Tr1 subset accumulates in an IL-6-and IL-10-dependent manner [35]. However, lowered T cell numbers, especially the CD4^+^ subset, have also been reported during aging, and after chronic hepatitis C infection and MS [36,37]. This decrease has been associated with an impaired immune response, rendering patients more susceptible to viral infections and the development of autoimmunity [36,38]. In a mouse stroke model, lymphopenia during the first week after injury was associated with decreased INF-γ levels and increased susceptibility to bacterial infections [24].

Previously, it has been shown that progression of DCM can lead to an increased inflammatory response by the recruitment and activation of different immune cells (i.e., microglia/macrophages, T cells and neutrophils) into the spinal cord [9,10]. In the current study, we show that during progression of DCM the frequency of circulating white blood cells is modestly affected over time, suggesting that white blood cell monitoring may not be a valuable tool for long-term monitoring of DCM progression. Since immune cell frequencies were normalized to matched naïve mice, we conclude that the above differences likely reflect a dysregulated immune response in the animals with DCM due to compression. Notwithstanding the clinical relevance of our model, similar studies in patients with DCM are needed to confirm if this is the case and further explore whether our observations are species-specific or sex specific.

Our study has some limitations. Although we measured the production of peripheral cytokines, we did not assess the expression of such cytokines in the circulating immune cells assessed here. This not only would more accurately match cell frequency with cytokine expression levels, but also it could provide information about the activation state of the immune cells during DCM. Secondly, changes in circulating white blood cells were assessed in a mouse model, so future studies should address whether similar findings are observed in DCM patients.

Taken together, the current study provides a basis for a deeper understanding towards the progression of DCM, by assessing the changes in the composition of circulating white blood cells. Similar longitudinal studies in patients with DCM are warranted to establish relevant time points of hematological profiling and disease-specific references. A better characterization of the systemic immune profiling in DCM allows surgeons to prepare for unexpected responses that might deviate from the normal time course and thus guide appropriate clinical decision-making. Furthermore, DCM-specific references for systemic white blood cells could be useful for monitoring patient responses to potential neuroprotective peri- or post-operative treatments for DCM patients. This is of special interest, for optimal administration of glucocorticoids following surgery or to decide optimal timing of surgical decompression.

## 4. Materials and Methods

### 4.1. Induction of DCM

Adult 8-weeks-old C57BL/6 female mice were acquired from the Ontario Council Institute (Canada) and ISP (Chile). DCM was induced by implanting an aromatic polyether material underneath the C5–C6 laminae, leading to a progressive compression of the cervical spinal due to osteoid formation between the aromatic polyether material and the laminae [11]. Two control groups were included, a sham and a naïve age-matched group. In the sham group, the aromatic polyether material was inserted under the C5-6 laminae, without damaging the spinal cord or performing laminectomy, for 30 s and then removed (Figure 1A). A detail of the experimental groups and the animals allocated for each assessment is shown in Figure 1A. All surgical procedures were performed under anesthesia using 2% isoflurane. Animals were sacrificed at 12 weeks after DCM or sham operation by excess isoflurane. This study was approved by the Animal Use Committee (AUC) of the University Health Network (UHN) in Toronto, Ontario, Canada, and the AUC of the Universidad Católica de la Santísima Concepción in Concepcion, Chile. All experiments were carried out in accordance with AUC guidelines.

All evaluations were performed by one or two investigators that were blinded to the treatment groups.

### 4.2. Flow Cytometric Analysis of Hematological Changes

Longitudinal blood sampling of mice was performed via saphenous vein puncture without anesthesia. Blood samples were collected in tubes coated with Ethylenediaminetetraacetic acid (EDTA) as anti-coagulant using a 23 G needle for the puncture [39]. Samples were collected at 3, 6, 9 and 12 weeks after DCM induction and analyzed by flow cytometry as previously described (Figure 1A) [11,40]. The following antibodies were used to distinguish granulocytes, monocytes and T lymphocytes in blood: Ly6C (clone HK1.4), Ly6G (clone 1A8), CD11b (clone M1/70), CD3 (clone 17A2), all from BioLegend (San Diego, CA, USA). Matching isotype controls were used for gating of the positive cells. Data were acquired using a BD LSR II flow cytometer (BD Biosciences, Mississauga, ON, Canada) and BD FACS Canto II (BD Biosciences, San Jose, CA, USA) analyzed using FlowJo × 10 (BD Biosciences, Ashland, Wilmington, DE, USA). The results from DCM and sham groups were normalized to the naive age-matched group. The results were acquired from 3 independent experiments (groups 1, 3 and 4, Figure 1A). Of note, a maximum of 3 attempts were made for blood collection from the saphenous vein [41]. Thus, the differences in group size between each time point are due to low blood volume collection for some time points.

### 4.3. Luminex xMAP Assay for the Quantitation of Cytokines in Serum

At 12 weeks post-DCM induction, mice were anesthetized using isoflurane and blood samples were collected via cardiac puncture before transcardial perfusion (Figure 1A). Blood samples were left to coagulate for 30 min at room temperature (RT) and then centrifugated at 3000 rpm for 5 min. Next, the collected serum samples were stored at −80 °C until used for analysis. A mouse 10-Plex Luminex array was used to quantitate levels of selected cytokines in the collected serum samples (Eve Technologies, Calgary, AB, Canada). Values for IL-12p70 and IL-β were out of range and were therefore removed from further analysis. Samples whose results were out of range were removed from the study (group 1 and 2 were used for this analysis, Figure 1A).

### 4.4. Histological Assessment

At 12 weeks post-DCM induction mice were euthanized using isoflurane overdose, and transcardially perfused with 4% paraformaldehyde (PFA). The spinal column was dissected out and immersed in Decalcifying Solution-Lite (Sigma-Aldrich, Saint Louis, MO, USA) for 3 days according to manufacturer instructions. Spinal column samples were embedded in OCT solution and sliced into 30 μm sections on a cryostat. The spinal column and spinal cord samples were stained by HE and imaged on a Zeiss Axioplan2 epifluorescence microscope (Zeiss, Oberkochen, Germany) at the epicenter of compression (Figure 1B). For NeuN staining, coronal spinal cord sections (30 μm) were blocked in 10% non-fat milk, 1% BSA, 0.3% triton X-100 in PBS for 1 h at RT, followed by incubation for 1 h with NeuN conjugated to Alexa-Fluor 555 (1:250, clone A60, Millipore) in combination with with 4′, 6-diamidino-2-phenylindole (DAPI; 1:200) at RT. Images were acquired using a Nikon eclipse Ti C2+ inverted confocal microscope.

### 4.5. Horizontal Ladder Walk to Assess Neurological Deficits

The ladder walk consisted of two plexiglass plates (69 × 9 cm) connected by 10 rungs, each 1 cm apart as previously described [42]. Animals were acclimatized to the ladder walk a week before the assessment, which took place at 12 weeks post-DCM induction. Two trials were video-recorded for each animal, and the foot fault steps out of all steps during each trial were counted [42]. The average (of two trials) percent (%) foot fault steps were plotted for each animal (group 1 was used for this analysis, Figure 1A).

### 4.6. Catwalk to Assess Overground Locomotor Behavioral

The Catwalk XT walkway system (Noldus) was used to assess overground gait parameters post-DCM induction as previously described [11,19]. The animals were allowed to freely walk across the walkway while being recorded. The following parameters were analyzed: swing speed, stride length and basal support in sham and DCM groups at 4, 6, 10, and 12 weeks after DCM induction (group 2 was used for this analysis, Figure 1A).

### 4.7. Statistical Analysis

The results were analyzed using Prism 9.0 (GraphPad Software, La Jolla, CA, USA) software. CatWalk results were analyzed using either a one-way ANOVA or a Two-Way ANOVA with Tukey’s correction. The horizontal ladder walk and cytokine levels were analyzed using Mann–Whitney U test. All data are presented as mean ± standard error of the mean (SEM). Two-tailed *p*-value < 0.05 indicated statistical significance. Flow cytometry results were assessed using a Mixed-effects model (or two ways repeated ANOVA), with the Geisser–Greenhouse correction followed by pairwise Sidak’s post-hoc tests, as needed. All data were tested for normal distribution using the Shapiro–Wilk test. Effect size (Hedge’s g) for every analysis in this study, was estimated using the online tool https://www.estimationstats.com accessed on 1 August 2019.

## Data Availability

The datasets used and/or analyzed during the current study are available from the corresponding author on reasonable request.

## Abbreviations

Central nervous system: CNS; Degenerative cervical myelopathy: DCM; Ethylenediaminetetraacetic acid: EDTA; Hematoxylin and eosin: HE; Multiple sclerosis: MS; SEM: Standard error of the mean; Spinal cord injury: SCI.
